# Focused cardiac ultrasound by unselected residents—the challenges

**DOI:** 10.1186/s12880-017-0191-y

**Published:** 2017-03-04

**Authors:** Vidar Ruddox, Ingvild Billehaug Norum, Thomas Muri Stokke, Thor Edvardsen, Jan Erik Otterstad

**Affiliations:** 1 Department of cardiology, Vestfold Hospital Trust, Po. Box 2168, N3103 Tønsberg, Norway; 2 Department of cardiology, Oslo University Hospital, Rikshospitalet, and University of Oslo, Oslo, Norway; 3 Center for Cardiological Innovation, Oslo University Hospital, Rikshospitalet, Oslo, Norway

## Abstract

**Background:**

Focus Cardiac Ultrasound (FoCUS) performed by internal medicine residents on call with 2 h of training can provide a means for ruling out cardiac disease, but with poor sensitivity. The purpose of the present study was to evaluate diagnostic usefulness as well as diagnostic accuracy of FoCUS following 4 h of training.

**Methods:**

All residents on call were given a 4-h training course with an additional one-hour training course after 6 months. They were asked to provide a pre- and post-FoCUS diagnosis, with the final diagnosis at discharge as reference.

**Results:**

During a 7 month period 113 FoCUS examinations were reported; after 53 were excluded this left 60 for evaluation with a standard echocardiogram performed on average 11.5 h after FoCUS. Examinations were performed on the basis of chest pain and dyspnoea/edema. The best sensitivity was found in terms of the detection of reduced left ventricular (LV) ejection fraction (EF) (92%), LV dilatation (85%) and pericardial effusion (100%). High values were noted for negative predictive values, although false positives were seen. A kappa > 0.6 was observed for reduced LVEF, right ventricular area fraction and dilatation of LV and left atrium. In 48% of patients pre- and post-FoCUS diagnoses were identical and concordant with the final diagnosis. Importantly, in 30% examinations FoCUS correctly changed the pre-FoCUS diagnosis.

**Conclusions:**

A FoCUS protocol with a 4-h training program gained clinical usefulness in one third of examinations. False positive findings represented the major challenge.

## Background

Cardiac ultrasound by pocket-size imaging devices has been extensively studied as an adjunct to physical examination at the bedside, both in emergency situations [1–3] and in point of care decisions [4–6]. In 2014, the European Association of Cardiovascular Imaging (EACVI) issued a position paper describing the use of Focus cardiac ultrasound (FoCUS) [7]. This term describes a point-of-care ultrasound examination performed according to a predefined restricted scanning protocol. Both cardiologists and non-cardiologists can perform FoCUS, with the protocol not restricted to specific equipment such as the pocket-size device [8]. It was stressed that a FoCUS exam is *not* to be referred to as an ordinary echocardiographic study and that specific training and accreditation procedure has to be constituted and furthermore, that scientific bodies or specialist organizations have to define when FoCUS may be used.

There are, at present, no general guidelines for training, competence and safe utilization of pocket-size imaging device FoCUS by non-cardiologists, including residents in internal medicine. In a recent study from our department on the implementation of pocket-size cardiac ultrasound among residents with a 2 h training protocol the FoCUS provided a means for ruling out significant disease [9]. At that time, encouraging results based upon such a short training had been reported for non-cardiologists using laptop-sized devices [10]. A firm impression, however, was that more training was needed for improvement of the poor diagnostic sensitivity observed.

The present study was undertaken to investigate the clinical usefulness of FoCUS by unselected residents working on call in a medical department after an a priori defined 4 h training protocol, and further to examine its diagnostic accuracy.

## Methods

This is a cross-sectional study following the entire staff of residents in an internal medicine department performing FoCUS after completing a predefined training period. Written informed consent was obtained from all patients and residents participating in the study, which has been approved by the Regional Committees for Medical and Health Research Ethics in Norway (2014/152) and by the Patient Ombudsman at the Norwegian Social Science Data Services (38382 / 3 / LT).

### Study protocol

FoCUS examination of patients was performed as an adjunct to the physical examination by participating residents according to a predefined protocol (Table 1). FoCUS was to be performed primarily upon patient admission (but also in hospitalized patient acute scenarios) at the resident’s discretion when satisfying one of four indications; either chest pain, murmur, dyspnea/edema or suspected pericardial effusion. A tentative diagnosis had to be suggested both before and after FoCUS.

**Table 1:** Cardiac parameters and their evaluation by focus cardiac ultrasound (FoCUS) and Standard Echocardiogram (SE)

	FoCUS	Standard Echocardiogram
LV EF (<40%^a^)	Visually (no/yes), A4C, LAX	Volume calculations using the biplane method of disks (modified Simpson’s rule) [17, 21, 22]
LV dilated		
LV WMA	Visually (no/yes), A4C, LAX, A2C	
Pericardial effusion	Calliper > 5 mm in end-diastole in any view. (no/yes)	
Aortic regurgitation^b^	Visual assessment of the jet area (no/yes) [22].	Predominantly by vena contracta measurements. Additionally, the pressure half time method was incorporated when grading AR. [18–20]
Mitral regurgitation^b^		
RV FAC (<30%)	Visually (no/yes), A4C	FAC in A4C [17]
RV dilated ^c^		Single plane area measurements in A4C [17]
RV WMA		Visually (no/yes), A4C
Aortic dilation	Calliper ≥ 4.0 cm, (no/yes)	
LA dilated^c^	Visually (no/yes), A4C	Biplane area-length method from A4C and LAX [17, 23]

*LV* left ventricle; EF ejection fraction, *WMA* wall motion abnormalities, *RV* right ventricle, *FAC* fractional area change, *LA* left atrium, *FoCUS* focus cardiac ultrasound, *SE* standard echocardiogram, *A4C* apical 4-chamber view, *LAX* apical long axis view, *A4C* apical 2-chamber view, *CW* continuous wave Doppler, *ASE* American Society of Cardiology, *AR* aortic regurgitation

^a^Visual estimate of LV systolic function on basis of EF being over or below 40% This arbitrarily chosen level was in accordance with current heart failure guidelines [13]

^b^Moderate or severe

^c^Dilated if more than half the area as compared to that of the LV

Following FoCUS a standard echocardiography (SE) was performed (blinded to the FoCUS results) by one of two level III echocardiographers [11, 12]. Written informed consent was obtained prior to SE. The SE examination was performed at the earliest opportunity the following weekday.

Exclusion criteria for the reference SE were lack of informed consent, resident knowledge of an echocardiogram performed within 3 months prior to index FoCUS, more than 48 h had passing between FoCUS and SE, if significant hemodynamic changes had occurred between FoCUS and SE (decided by a consensus in the study group), or if the patient had been discharged before a SE could be performed.

### Echocardiography

The FoCUS examinations were performed using a Vscan pocket-size imaging device [8] defined as the index test. The SE was defined as the reference test, and was performed with the Vivid E9 scanner (both: GE Vingmed Ultrasound, Horten, Norway).

The principles applied for assessment of the cardiac structures with the two imaging modalities are shown in Table 1. Of note, the pocket-size imaging device only allows for the qualitative assessment of most structures, albeit that simple calliper measurements can be performed. Priority was given to left ventricular (LV) systolic function (ejection fraction (EF) over or below 40%, chosen due to the status of current heart failure guidelines [13]) and size, as well as the finding of pericardial effusion or not. If visualization of the LV was not possible such that assessment was unfeasible, the examination was discarded and the patient was not subjected to a reference SE.

### Residents and FoCUS training

All residents participated in an intensive training program before entering the study. Intensive training was defined as 4 h of highly intense teaching in small participant groups. In addition, they were given an additional one-hour recertification course after approximately 6 months.

Training material was handed out prior to the initial course with information about echocardiographic views, qualitative assessment and indications for FoCUS as well as links to training sites and video recordings of normal and pathological findings. The residents were encouraged but not required to study prior to the course, and no post-test component to this pre-course module was applied. Training courses were performed with 2–4 residents, consisting of a 45-min introduction with a presentation of video-loops of cardiac parameters evident in Table 1.

For simplicity, residents were taught to record a single parasternal (long axis) and apical (4-chamber) cineloop with grey scale and color Doppler flow imaging. Cardiac parameters and method of evaluation is described in Table 1 (the FoCUS column). A practical walk-through of a Vscan operation to obtain these views was given in addition to a 60-min hands-on session where residents practiced on each other under supervision. This was followed by a 90-min bedside supervised hands-on practice in pairs on unselected patients in our department of cardiology. An instructor was present in this hands-on session, going through a checklist of views, indices and interpretations corresponding to that presented in Table 1. No formal testing of competence was applied.

The recertification course presented the residents with two patients recruited from ward without any selection criteria applied. The resident passed if the echocardiographic windows described previously with relevant cardiac structures could be visualized and commented on in a normal/pathologic fashion. As in the pre-study course, an instructor was present and no formal testing of competence was applied.

### Setting

The study was performed in the Medical department at Vestfold Hospital Trust in Norway; a medium sized hospital without invasive cardiology service. The emergency department handles acute admissions from the county of Vestfold (catchment area 240 000 inhabitants), referred from general practitioners as well as pre-hospital medical services.

### Diagnostic usefulness of FoCUS versus the initial clinical examination

**Fig. 1:**
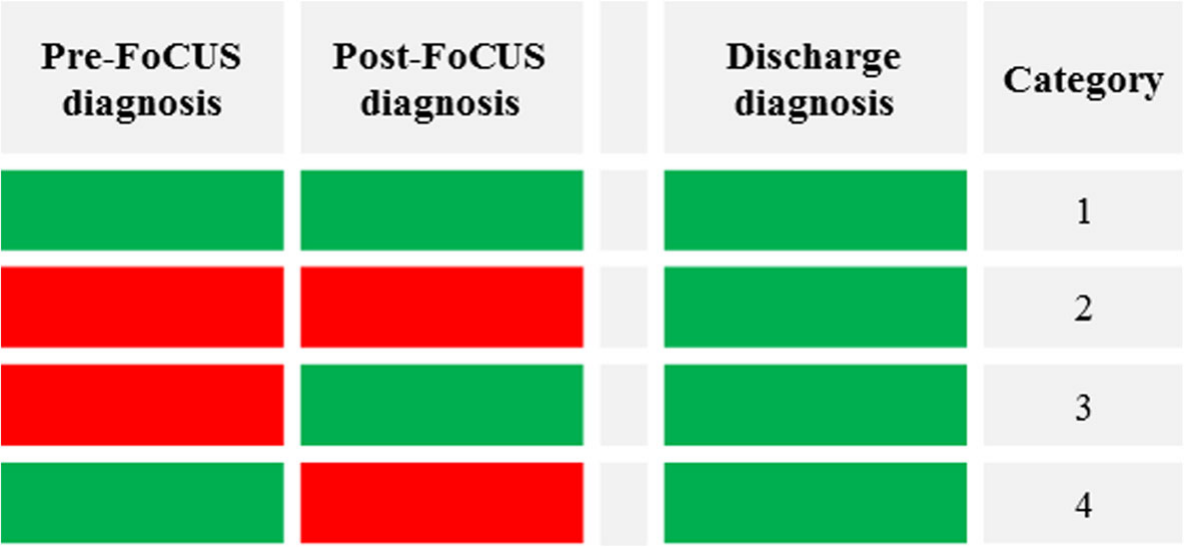
For each of the four categories defined to study diagnostic usefulness we have depicted the concordance (boxes equally marked) or discordance (boxes not equally marked) of diagnoses set pre-FoCUS, post-FoCUS and at discharge. In category 1 and 2, no diagnostic usefulness is observed as pre-FoCUS diagnosis is not changed. In category 3, diagnosis is changed correctly on the basis of a FoCUS examination. Finally, in category 4 the diagnosis is erroneously changed from the correct pre-FoCUS diagnosis

The diagnostic usefulness of bedside FoCUS was evaluated from the preliminary diagnosis following the initial clinical examination (pre-FOCUS) and then after the pocket-size cardiac ultrasound evaluation (post-FoCUS), both given by the residents, using the final discharge diagnosis as reference. Four comparative categories for the utility of FoCUS were created as depicted in Fig. 1:

1. Identical pre- and post-FoCUS diagnoses, being concordant to the discharge diagnosis
  This category would describe cases where FoCUS bears no diagnostic influence as well as cases where the initial tentative diagnosis was verified by FoCUS.
2. Identical pre- and post-FoCUS diagnoses, being discordant to the discharge diagnosis
  This category would also describe cases in which FoCUS had no diagnostic influence but also cases where a differential diagnosis was ruled out.
3. Discordant pre-focus and concordant post-FoCUS diagnosis
  Category 3 would describe cases where FoCUS yield high diagnostic usefulness; the diagnosis was changed and shown to match the discharge diagnosis.
4. Concordant pre-FoCUS and discordant post-FoCUS diagnosis
  In such cases the initial (pre-FoCUS) diagnoses was correct but the FoCUS examinations provided an erroneous diagnosis. This category represents the erroneous examinations that may be potentially harmful to the patient.

The reason for introducing these diagnostic categories was to use them as a marker for clinical usefulness, where examinations categorized in group 3 and 4 would have a great impact on patient management.

### Statistics

Statistical analyses were performed using SPSS for Windows, version 21 (IBM SPSS Statistics, IBM Corporation, Armonk, NY, USA). A Shapiro-Wilk test was used to test variables for the assumption of normality. Continuous variables with a near normal distribution are presented as either mean followed by standard deviation (SD) or range where appropriate, whereas data with a skewed distribution is presented as median followed by interquartile range (IQR). Categorical data is presented in terms of proportions. An independent samples *t*-test was used for the comparison of normally distributed continuous data, while for skewed continuous data an independent sample Mann–Whitney *U* test was used. Proportions were analyzed using a chi-square test. Two-tailed p-values lower than 0.05 were considered statistically significant.

As the residents were only active in the study from the conclusion of training to the closure of the study, active participating time was calculated for each resident. To find the true timespan of the study, the times of all participants were added and divided by the number of residents. If a resident quit or was given other assignments without such duty, the resident would no longer participate in the study. No sample size calculation was performed given that this was a convenience sample study.

Diagnostic accuracy was reported as sensitivity, specificity and positive/negative predictive values (PPV/NPV) and was calculated for each diagnosis using binary no/yes variables and is presented as percentage (95%CI). Agreement was calculated using Cohen’s Kappa coefficient for inter-rater agreement (*k*) for categorical variables. Kappa values of less than 0.2 were interpreted as ‘slight’ agreement, 0.21 to 0.4 as ‘fair’, 0.41 to 0.6 as ‘moderate’, 0.61 to 0.8 as ‘good’ and 0.81 to 1.00 as ‘very good’.

## Results

**Fig. 2:**
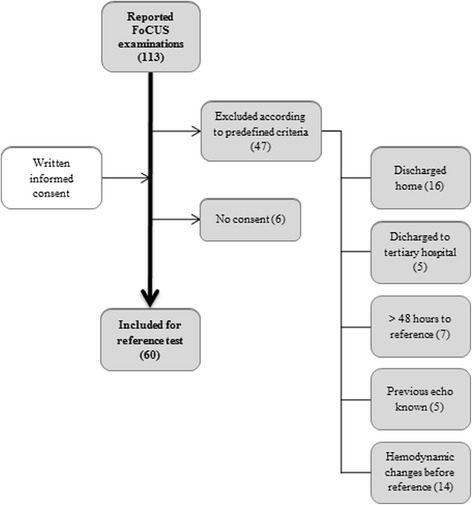
A flow chart showing patients screened with FoCUS subsequently found to be eligible for reference standard examination. Excluded patients have been stratified according to the reason for their exclusion

Figure 2 depicts the study flow of the 113 reported FoCUS-examinations during the study period. The most frequent reasons for exclusion (*n* = 47) were that patients had been discharged (*n* = 16) or had significant hemodynamic changes (*n* = 14) before the SE was available. A further 6 did not give their consent to participate. The characteristics of the 60 patients included are shown in Table 2. Most FoCUS-examinations, in both included and excluded patients, were performed on the basis of either chest pain or dyspnea/edema as shown in Table 3.

**Table 2:** Characteristics of the study population

	Included
N (%)	
Number of cases	60
Male	43 (72)
Atrial Fibrillation	14 (23)
Median (IQR)	
Age (years)	74 (55–81)
BMI (kg/m^2^)	24.7 (22.3-27.7)
Heart Rate	85 (73–99)
Systolic BP (mmHg)	131 (112–149)
Diastolic BP (mmHg)	76 (62–85)

*N* numbers, *IQR* interquartile range, *BMI* body mass index, *BP* blood pressure

**Table 3:** Reported clinical symptom/symptom combination necessitating a focused cardiac ultrasound (FoCUS) examination

	Included, n (%)	Excluded, n (%)
Dyspnea/Edema	29 (48)	24 (40)
Chest Pain	25 (42)	24 (40)
Murmur	1 (2)	1 (2)
Pericardial effusion	5 (8)	4 (8)

*N* number

All 24 residents gave their written informed consent to participate in the study and received the scheduled FoCUS training during the study period. Active participation time was 6.8 (range 4–9) months. Four residents did not produce any examinations that were evaluated in the study; two chose not to use FoCUS at all and another two had all their FoCUS-examinations (*n* = 7) excluded due to the fact that we were not able to perform a reference test (5 patients were discharged before SE could be performed and two had significant hemodynamic changes). The remaining 20 reported a range of 1–7 examinations.

Median (IQR) time for FoCUS was 8 (5–10) minutes and the SE was performed 11.5 (4.2-19.5) hours after FoCUS.

Table 4 shows number of registrations per parameter and prevalence of abnormal findings with each method as well as the diagnostic accuracy in terms of sensitivity, specificity, positive predictive value (PPV), negative predictive value (NPV) and agreement.

**Table 4:** Results from focus cardiac ultrasound (FoCUS) examinations as performed by internal medicine residents with minimal training. Validated by a Standard Echocardiogram (SE)

	FoCUS & SE N_registrations_	FoCUS N_abnormal_	SE N_abnormal_	Sens (95% CI) %	Spes (95% CI) %	PPV (95% CI) %	NPV (95% CI) %	k
LV EF (<40%)	60	29	24	92 (72–99)	81 (63–91)	76 (56–89)	94 (77–99)	0,70
LV dilated	56	13	13	85 (54–97)	100 (89–100)	100 (68–100)	96 (84–99)	0.75
LV WMA	59	23	21	70 (46–87)	78 (60–88)	61 (39–80)	83 (67–93)	0.44
Pericardial effusion	57	11	3	100 (31–100)	85 (72–93)	27 (7–61)	100 (90–100)	0.30
Aortic regurgitation	43	7	10	40 (14–73)	91 (74–98)	57 (20–88)	83 (67–93)	0.35
Mitral regurgitation^a^	52	7	9	56 (23–85)	95 (83–99)	71 (30–95)	91 (77–97)	0.56
RV FAC (<30%)	45	6	8	50 (14–86)	97 (84–99)	75 (22–99)	92 (78–98)	0.66
RV dilated	52	10	6	67 (24–94)	87 (73–95)	40 (14–73)	95 (83–99)	0.42
RV WMA	44	2	2	50 (2–97)	98 (86–100)	50 (2–97)	98 (86–100)	0.48
Aortic dilation	37	0	2	0 (0–12)	100 (88–100)	n.a.	95 (81–99)	0.0
LA dilated	47	18	24	71 (49–87)	96 (76–100)	94 (71–100)	76 (56–89)	0.66

*N* number, *k* Cohen’s kappa for inter-rater agreement, *Sens* sensitivity, *CI* confidence interval, *Spec* specificity, *PPV* positive predictive value, *NPV* negative predictive value, *LV* left ventricle; *EF* ejection fraction, *WMA* wall motion abnormalities, *RV* right ventricle, *FAC* fractional area change

^a^moderate or severe

Table 4 shows the number of registered FoCUS examinations of each parameter, n abnormal findings by FoCUS and SE, the respective sensitivity, specificity, positive predictive and negative predictive value for detection of cardiac pathology by FoCUS and kappa value for inter-rater agreement between the two methods

Sensitivity for LV EF <40% and dilation as well as the detection of pericardial effusion was 85% or more. The PPV for the detection of dilated LV was strong. Overall high specificities and NPVs were seen, with the majority of NPVs being above 90% excluding LV wall motion abnormalities, aortic regurgitation and dilated left atrium. ‘Good’ agreement was apparent for the detection of LV EF <40%, a dilated LV, RV fractional area change <30% and a dilated left atrium. For all other indices, agreement was either slight (*n* = 1), fair (*n* = 2) or moderate (*n* = 4).

Low sensitivity and PPV were seen for left sided valve regurgitations, RV indices and aortic dilation. Sensitivity for detecting pericardial effusion was high albeit with very low PPV indicating false positives. With the LA, a number of false negatives were seen as reflected by the low NPV.

Nearly two thirds (66%) of the pre- and post-FoCUS diagnoses were concordant with the discharge diagnosis (category 1 and two as presented in the methods and Fig. 1). Approximately one third (30%) of the examinations were correctly changed after FoCUS and thus are placed in diagnostic category 3 whereas a small percentage (4%) were erroneously changed and comprise category 4.

## Discussion

In the present study we found a favourable diagnostic usefulness of FoCUS as a measure of clinical usefulness in one third of examinations, with no significant impact in nearly two thirds of examinations. Very few potentially harmful impacts on diagnostics were seen; in two cases FoCUS erroneously changed the tentative diagnosis. Furthermore, NPVs for LV indices (except WMA), as well as pericardial effusion, RV indices and aortic dilation were high and we also found strong PPV to detect dilated LV and LA. All other indices studied showed low PPVs, indicating a number of false positives.

We therefore believe that in the initial patient assessment FoCUS can be used as an additional diagnostic tool for ruling the out of a limited number of cardiac diagnoses. Whether or not FoCUS should be used in the ruling *in* of cardiac diagnoses remains questionable.

Evaluating the accuracy of individual components of a FoCUS examination is methodologically problematic, particularly if the "best" results are then picked out. Yet, it is insightful to do so since there is no strong consensus as to which should be included in a FoCUS examination following a very limited training. Hence, accuracies presented in Table 4 indicate that the evaluation of RV might not be as feasible as LV evaluation in this context.

The two cases where FoCUS had a potentially harmful impact have to be scrutinized. The first was a chest pain patient with dyspnea suspective of acute coronary syndrome. FoCUS showed normal LV function but the resident suspected RV pathology and pulmonary embolism. The diagnostic workup ruled out any pulmonary pathology and a subsequent coronary angiogram revealed three vessel coronary heart disease. In the second case a patient was referred with dyspnea suspect of pulmonary pathology. FoCUS examination was reported as normal, which later was verified by the SE. However, the diagnostic workup confirmed peripheral pulmonary emboli. It is of note that low-risk pulmonary emboli, as in this case, is usually not associated with RV dysfunction, but this exemplifies the potential harm a pocket-size ultrasound device can facilitate in the hands of the minimally trained.

Two findings of clinical significance need further attention. First, a dilated LA may reflect chronic LV diastolic dysfunction in cases of a preserved LVEF and therefore accordingly give support to a diagnosis of diastolic heart failure. In cases of a mitral regurgitation, a dilated LA may give additional information as to its severity. Furthermore, the size of LA may be of value in the prognostic assessment and management of patients with atrial fibrillation. Secondly, the finding or exclusion of pericardial effusion is also clinically relevant, given the possibility of quantification and evaluation for eventual pericardioscentesis.

We did not report to what extent FoCUS was used but not reported. Based upon personal communication with our residents, underreporting has certainly taken place. The reasons given were, apart from poor image quality or examinations not possible to interpret, a lack of prioritization given the time needed for fill out additional reports during a busy working practice.

In our first study of FoCUS we identified the same infrequent use by many of the residents [9]. We have now reduced the number of indications and outlined potential pitfalls on the basis of previous results and experience, such that the current utilization is different to previously. As opposed to the preceding study, where 303 examinations could be evaluated during a similar study period, the participants now decided for themselves whether their recording could be regarded as good enough for diagnostic purposes and for the subsequent SE evaluation. This may indicate a selection bias of good quality recordings, which again may explain the infrequent recordings with, albeit, improved results. This questions the generalizability of our results, but nevertheless one could argue that it reflects a real life scenario.

It is generally accepted that ‘practice makes better’, which is exemplified by Prinz et al. [14]. In this study, one cardiology fellow with no echocardiographic skills (except from a basic introduction on performing cardiac ultrasound) trained for 8 weeks and undertook 40 examinations per week (plus two sessions per week under the supervision of an expert), which resulted in an improvement in image quality and agreement with SE over time. The limited use of FoCUS by the residents might have compromised the results in terms of diagnostic accuracy due to low volume practice.

The large number of excluded examinations partly explained by the time delay observed between FoCUS and SE, may have influenced the results. These events were clearly defined as exclusion criteria beforehand, and do not necessarily represent a systematic source of bias. However, examinations providing a diagnostic result could have improved sensitivity and PPV further, exemplified by patients being transferred to a tertiary hospital for an acute coronary syndrome before SE could be performed, where the FoCUS examination had given rise to the suspicion of coronary occlusion needing invasive management.

The diagnostic strong NPV in the present study was also reported in the study of Mjølstad et al. [3]. In that study, one half of the entire staff of internal medicine residents working on-call (6/12) was randomized to receive a 4 h lecture and 3 months of practice training. After excluding patients who did not give their informed consent and those having been discharged before reference SE, they were able to include 199/446 patients. NPV >90% was reported for right and left ventricular function and >80% for left sided valvular heart disease. Furthermore, sensitivity and PPV for LV dysfunction were similar to our results (92% and 80% respectively).

On the other hand, Galderisi et al. [5] reported a lower specificity (72%) than sensitivity (87%) for non-expert FoCUS. In that study no predictive values were presented and parameter-specific results were only presented with kappa agreement, not sensitivity and specificity. Patients were selected on the basis of good quality imaging and those with overt heart failure, a history of coronary artery disease, recognized valvular heart disease or primary cardiomyopathies were excluded. To what extent non-experts were selected or not was not reported, but they comprised an unknown number of residents in internal medicine who had been through a very comprehensive training (15 h lectures and 150 examinations).

The results we present for clinical usefulness are somewhat reassuring given the few erroneous alterations of a correct diagnosis. Additionally, the number of cases where the initial diagnosis was changed correctly following FoCUS is reassuring for safe utilization of the method.

Clinical impact has also been reported by Panoulas et al. [2] as an increase in sensitivity for various cardiac disorders from 26% by physical examination alone to 74% after adding FoCUS. Five selected medical students and three junior doctors who had completed a 2 h standardized training participated. FoCUS as performed by a cardiologist was used as reference.

The patients comprising the study population were commonly presenting with chest pain and dyspnea/edema, were elderly, normotensive and ¼ had atrial fibrillation. The patients presented in our study therefore represent a very common cross-section of patients referred to a general hospital.

### Limitations

We have presented FoCUS examinations that are biased by selection, both in terms of operator, image and patient. Motivated operators were prone to report good quality images of patients that were easy to examine, all being compatible with selection bias.

The feasibility of FoCUS was not reported in this study. However, it has been reported by others, varying from 96% to 100% [3, 15, 16] in terms of the ability to visualize the LV (amongst other indices). In a pilot study, using a 2 h teaching program described elsewhere [9], we found the feasibility to visualize the LV to be 86%. Additionally, we had to exclude a large number of examinations due to administrative and medical factors. A lower proportion of excluded examinations might have been achieved if all SEs could have been performed directly following the FoCUS. Furthermore, this was a convenience sample study.

The selection described above led to few included cases when seen in relation to the large number of residents, excluded examinations and the timespan of the study. However, it is of note that the residents were not active for the entire period; on average they were active for less than seven months.

We have compared FoCUS performed with a pocket-size device containing no advanced measure capabilities other than linear distances (and performed by staff with limited training) to advanced echocardiographic machines operated by trained echocardiographers. These two ultrasound methods have profoundly different approaches for cardiac evaluation. Visual assessments are not regarded as sufficient for an SE according to contemporary recommendations [17–20]. On the other hand, visual assessment by a pocket-size device to complement the physical examination is one of eight stated uses for FoCUS [8], and after long debates on the so called eye-balling technique, we have chosen SE as the reference for FoCUS examinations.

The severity of pathology may also represent a bias as dichotomization collapses information. As an example, we studied LV WMA. This entity encompasses the distinction between four states of myocardial motion where, for instance, the distinction between hypo and normal kinesis is much more difficult than between akinesis and normal kinesis.

Taking the time delay from FoCUS to SE and predefined criteria for excluding studies into consideration, we cannot ignore the fact that significant changes may have taken place that could profoundly affect the measurements.

Intra- and inter-observer variations with FoCUS have not been studied. These entities have previously been extensively reported, including by our group [21].

## Conclusions

A FoCUS protocol with a 4-h training of unselected internal medicine residents was associated with clinical usefulness in one third of examinations. We generally found a good negative predictive ability for selected cardiac indices in patients presenting with chest pain and symptoms of heart failure. A generally low positive predictive value indicated a number of false positives. Future studies should aim to investigate in a randomized outcome trial the clinical impact of FoCUS compared with a physical examination alone.

## Abbreviations

EACVI: European Association of Cardiovascular Imaging
EF: Ejection fraction
FoCUS: Focus cardiac ultrasound
IQR: Interquartile range
LV: Left ventricle
NPV: Negative predictive value
PPV: Positive predictive value
SD: Standard deviation
SE: Standard echocardiography
